# Association of Sarcopenia and Its Defining Components with the Degree of Cognitive Impairment in a Memory Clinic Population

**DOI:** 10.3233/JAD-221186

**Published:** 2023-11-07

**Authors:** Liss Elin Larsson, Rui Wang, Tommy Cederholm, Fleur Wiggenraad, Marie Rydén, Göran Hagman, Mai-Lis Hellénius, Miia Kivipelto, Charlotta Thunborg

**Affiliations:** a Theme Inflammation and Aging, Karolinska University Hospital, Stockholm, Sweden; b The Swedish School of Sport and Health Science, GIH, Stockholm, Sweden; c Department of Neurobiology, Division of Clinical Geriatrics, Care Sciences and Society, Karolinska Institutet, Stockholm, Sweden; d Wisconsin Alzheimer’s Disease Research Center, University of Wisconsin School of Medicine and Public Health, Madison, WI, USA; e Department of Public Health and Caring Sciences, Clinical Nutrition and Metabolism, Uppsala University, Uppsala, Sweden; f Department of Medicine, Karolinska Institutet, Stockholm, Sweden; g Institute of Public Health and Clinical Nutrition, University of Eastern Finland, Kuopio, Finland; h The Ageing Epidemiology Research Unit, School of Public Health, Imperial College London, London, United Kingdom; i Mälardalen University Department of Health and Welfare, Sweden; j Department of Caring Sciences, Faculty of Health and Occupational Studies, University of Gävle, Sweden

**Keywords:** Alzheimer’s disease, body composition, cognitive function, gait speed, hand grip strength, outpatients, sarcopenia

## Abstract

**Background:**

Sarcopenia and cognitive impairment are two leading causes of disabilities.

**Objective:**

The objective was to examine the prevalence of sarcopenia and investigate the association between sarcopenia diagnostic components (muscle strength, muscle mass, and physical performance) and cognitive impairment in memory clinic patients.

**Methods:**

368 patients were included (age 59.0±7.25 years, women: 58.7%), displaying three clinical phenotypes of cognitive impairments, i.e., subjective cognitive impairment (SCI, 57%), mild cognitive impairment (MCI, 26%), and Alzheimer’s disease (AD, 17%). Sarcopenia was defined according to diagnostic algorithm recommended by the European Working Group on Sarcopenia in Older People. Components of sarcopenia were grip strength, bioelectrical impedance analysis, and gait speed. They were further aggregated into a score (0–3 points) by counting the numbers of limited components. Multi-nominal logistic regression was applied.

**Results:**

Probable sarcopenia (i.e., reduced grip strength) was observed in 9.6% of the patients, and 3.5% were diagnosed with sarcopenia. Patients with faster gait speed showed less likelihood of MCI (odds ratio [OR]: 0.24, 95% confidence interval [CI]: 0.06–0.90) and AD (OR: 0.12, 95% CI: 0.03–0.60). One or more limited sarcopenia components was associated with worse cognitive function. After adjusting for potential confounders, the association remained significant only for AD (OR 4.29, 95% CI 1.45–11.92).

**Conclusion:**

The results indicate a connection between the sarcopenia components and cognitive impairments. Limitations in the sarcopenia measures, especially slow walking speed, were related to poorer cognitive outcomes. More investigationsare required to further verify the causal relationship between sarcopenia and cognitive outcomes.

## 1 INTRODUCTION

Today’s global population of one billion people aged 60 and older will double in 30 years [1]. Age-related disorders linked to muscle and cognitive function leading to disability and dependency will increase [2, 3]. Sarcopenia and cognitive impairment are two main such disabling conditions [4, 5].

Sarcopenia is a progressive muscle disorder characterized by an accelerating loss of muscle mass and muscle function, i.e., loss of strength and performance [6]. According to the well-accepted diagnostic criteria of the European Working Group on Sarcopenia in Older People (EWGSOP2), the current diagnosis of sarcopenia is based on low measures of muscle strength, muscle mass, and physical performance [6]. The prevalence of sarcopenia is around 10% among community-dwelling older adults, with a steep increase with advancing age [7]. However, the prevalence of sarcopenia varies between studies and different populations attributed to a combination of factors related to study design, population characteristics, geographic and cultural influences, healthcare access, and evolving diagnostic criteria [8]. Furthermore, due to the aging population, the number of dementia cases worldwide is predicted to increase from 50 million to 152 million by 2050 [9, 10]. Alzheimer’s disease (AD) is the major cause of dementia and accounts for 60–70% of the cases [11]. The spectrum of AD spans frequently various stages, including normal cognition, mild cognitive impairments (MCI), and diagnosed dementia symptoms [12, 13]. Moreover, it is unclear if sarcopenia or memory problems occur first, and it is important to address that the phenomenon is prevalent both in younger and older adults.

Previous research has found that sarcopenia is associated with increased risk of AD [14, 15]. Specifically, a growing body of evidence suggests that reduced gait speed is associated with cognitive impairment [16–18]. Weaker grip strength is also associated with overall cognition and cognitive impairment [17, 19, 20]. These findings indicate that sarcopenia and dementia are closely linked, and may share common risk factors, as well as having mutual causal impact [14]. However, the data on the prevalence of sarcopenia in older adults with cognitive impairment, especially in a clinical setting, is insufficient [21–23]. This is extremely relevant to clinical practice and health care when facing older individuals with cognitive deficits, as sarcopenia and cognitive impairment can contribute to a downward spiral of functional decline and decreased quality of life in older populations. More importantly, investigating the prevalence of sarcopenia across distinct stages along the AD spectrum, subjective cognitive impairment (SCI), MCI, and the diagnosed AD phase, would significantly enhance our knowledge on the bi-direction relationships between sarcopenia and cognitive impairment. Therefore, exploring the potential occurrence of sarcopenia among patients within a real-world memory clinic becomes a source of intrigue, particularly given its relevance to the development of early intervention programs in a representative population [24].

Using data from a real-world memory clinic, patients referred to a specialized memory clinic this study aimed to examine the prevalence of sarcopenia and investigate the association between sarcopenia diagnostic components and cognitive impairment.

## 2 MATERIALS AND METHODS

### 2.1 Study design and participants

This cross-sectional study prospectively examined outpatients who attended the Memory Clinic in Solna at Karolinska University Hospital, Medical Unit Aging in Stockholm, Sweden. The specialized memory clinic accepts all individuals ≤70 years with cognitive complaints in the Stockholm region referred by general practitioners in primary and occupational health care in the catchment area (northern Stockholm). The memory clinic also accepts patients seeking a second diagnostic opinion regardless of age. Thus, in this analytical sample the age of patients ranges from 31 to 82 which is representative for this clinic. All diagnostic examinations are performed in a fast-track model within one week [25].

**Fig. 1:**
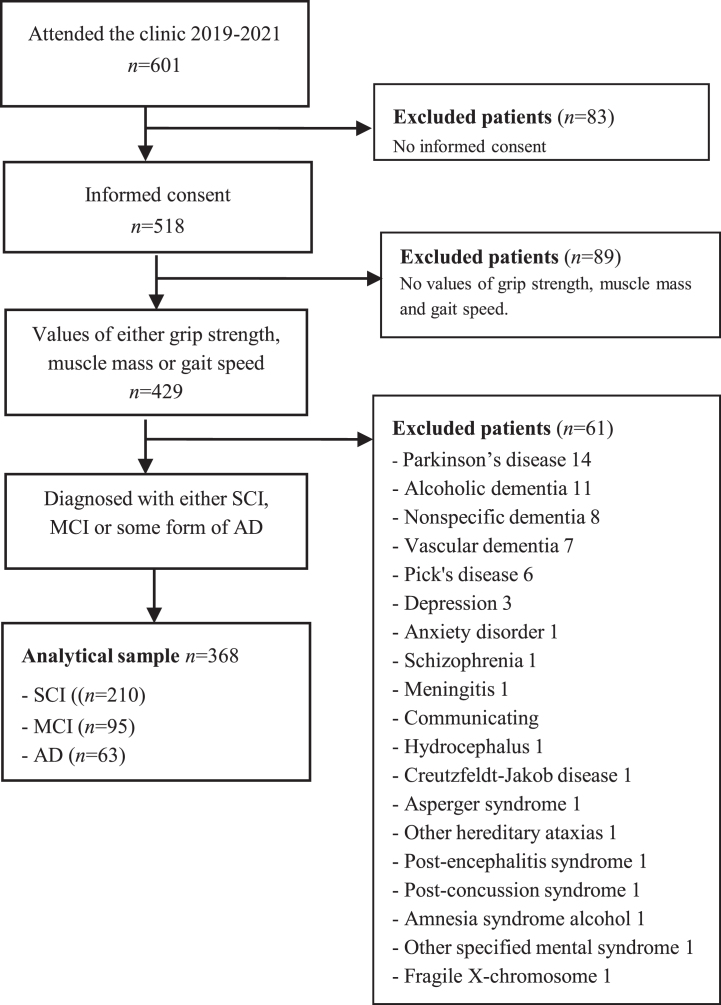
Flow chart of the study population.

All patients referred to the memory clinic are invited to participate, and after written informed consent, to include their clinical examination results in the electronic database and biobank for clinical research at the Geriatric Clinic at Karolinska University Hospital, i.e., the GEDOC database and biobank. The current study included patients who had their first diagnostic visit at the memory clinic between April 2019 and July 2021. All participants gave their written informed consent to be part of the GEDOC database, as well as of this study. Patients without any assessment of grip strength, muscle mass, or gait speed were excluded (N = 89), see Fig. 1. All patients were diagnosed with SCI, MCI, or AD. The GEDOC database, and the present study, have ethical approval, i.e., Dnr 2011-1978-31/4 and Dnr 2020-06484, provided by the Regional Ethical Review Board in Sweden. The need for GEDOC database patients to provide re-consent for their data’s inclusion in a future study is determined by the Ethical Review Board. In the context of this study, patients were not asked to re-provide their consent.

### 2.2 Neurological examination and dementia diagnosis

The patients undertook a comprehensive cognitive and functional capacity assessment. Prior to the referral, the patients completed a basic medical evaluation including medical history, brain imaging using magnetic resonance imaging (MRI), or computed tomography, and Mini-Mental State Examination (MMSE). At the memory clinic, all patients underwent a highly specialized medical and neurological evaluation. Medical and informant-based history, detailed neuropsychological testing, including Montreal Cognitive Assessment (MoCA), blood chemistry, *APOE* genotyping, MRI of the brain, and lumbar puncture to draw cerebrospinal fluid for collection of biomarkers are performed. Additional examinations are conducted based on clinical judgment, e.g., fluorodeoxyglucose (FDG)-PET, flutemetamol PET, and speech-language pathologist’s consultation. The dementia examination process follows national guidelines established by the Swedish Board of Health and Welfare [26]. A multidisciplinary team evaluates each patient and sets a consensus diagnosis based on all test results, including biomarkers. Diagnosis is based on the Diagnostic and Statistical Manual of Mental Disorders (DSM)-V criteria [27]. According to the DSM-V, dementia is termed major neurocognitive disorders and MCI mild neurocognitive disorders. Patients who do not meet MCI criteria or dementia are considered to have SCI.

### 2.3 Definition of sarcopenia

For this study, sarcopenia was defined according to the updated diagnostic algorithm by the European Working Group on Sarcopenia in Older People (EWGSOP2) [6]. The key characteristic of sarcopenia is low muscle strength, and the detection of low muscle quantity and quality confirms the sarcopenia diagnosis. In addition, severe sarcopenia is defined by poor physical performance. Accordingly, probable sarcopenia was identified by low muscle strength, and the diagnosis was confirmed by additional documentation of low muscle mass.

### 2.4 Muscle strength

Physical tests are performed on the first day of the clinical evaluation process by the physiotherapist (C.T.) to assess body composition and physical functions. These tests are thereby performed before diagnoses are set.

Handgrip strength was used to evaluate muscle strength and assessed by Jamar hydraulic dynamometer (Sammons Preston Inc.). The patient was standing with the back close to the wall and with shoulders adducted and neutrally rotated, elbow flexed to 90 degrees, and the forearm in a neutral position. Patients were instructed to squeeze the dynamometer with the dominant hand at their maximal effort. The test was performed three times. The average grip strength was calculated and recorded. Low muscle strength was defined as grip strength <16 kg for women and <27 kg for men [6].

The female and male patients were also grouped into tertiles according to gender-specific cut-offs, i.e., lowest tertile for females <21 kg, and for males <35 kg, medium tertile for females was 21–26.9 kg and for males 35–41.9 kg, and finally, highest tertile for females >27 and males ≥42 kg.

### 2.5 Muscle mass

Muscle mass was assessed by phase-sensitive multi-frequency bioelectrical impedance analysis (BIA) by Seca medical Body Composition Analyzer 515 (Seca mBCA 515, Seca, Hamburg, Germany). BIA is widely used to estimate muscle mass [28]. The examination was performed in a standing position according to the standard procedures, and each patient was scanned once. The modern BIA technique is a valid method, and the registration takes about 75 seconds. The value for fat-free mass index (FFMI) was used as a proxy measure of muscle mass, i.e., fat-free mass divided by height (meters squared (m^2^)). A threshold of FFMI <15 kg/m^2^ for women and <17 kg/m^2^ for men was used to determine low muscle mass [29].

Like for grip strength, all the female and male patients were classified into tertiles according to gender-specific cut-offs; the lowest tertile for females and males was <14.6 kg/m^2^ and <18.3 kg/m^2^, respectively. Medium tertiles for females and males were 14.6–15.9 kg/m^2^ and 18.3–19.89 kg/m^2^, respectively, and finally, the highest tertiles for females and males were ≥16 kg/m^2^ and ≥19.9 kg/m^2^, respectively.

### 2.6 Physical performance

Physical performance was assessed by gait speed using the Timed 10 meters walk test [30]. The mean of three tests was used for gait speed (m/s). Patients were instructed to walk a distance of 10 m (that was marked with tape on the floor) at a usual and comfortable speed. The walking time was measured with a stopwatch, and the test leader walked alongside the patient. Timing started when the patient’s foot crossed the 10 m start-mark and stopped when a foot crossed the 10 m end-mark. A gait speed ≤0.8 m/s was considered low physical performance [6].

Again, all patients were classified into tertiles according to gait speed, but this time with the same cut-off for both genders since gait speed is not expected to differ between older females and males, which is an observation-based commonly accepted assumption in the field of geriatrics [31, 32]. Thus, lowest tertile was <1.2 m/s; medium tertile 1.2–1.39 m/s, and highest tertile >1.4 m/s.

We have additionally performed a stratification analysis by the age of participants (≤70 years versus >70 years). The prevalence of sarcopenia was 3% in young patients (≤70 years) and 15% in older patients (>70 years). Please see more information in Supplementary Table 3.

### 2.7 Data analysis

Calculations were performed stepwise. First, all variables were checked for normality using visual assessment of histograms and normality tests. The prevalence of sarcopenia was calculated for each group (SCI, MCI, and AD) and compared using Fisher’s exact test due to the low frequency of sarcopenia, i.e., the number in all groups was <5. Differences between patients with and without sarcopenia were analyzed using Student’s *t*-test or Mann-Whitney U-test for continuous variables. The Chi-square test was used for categorical data. Differences in characteristics at various cognitive stages were analyzed using one-way ANOVA or The Kruskal-Wallis Test for continuous variables. The chi-squared test was used for categorical variables.

Furthermore, the association between cognitive impairment and the three components of sarcopenia (muscle strength, muscle mass, and physical performance) was assessed individually, as well as aggregated into a score (0–3 points) by counting the numbers of limited components. The three components were classified into tertiles as described above. The lowest tertile for each component was categorized as limited and generated a score of 1. Patients in the medium and highest tertile were classified as not limited and scored zero. The sum of limited components then generated a score of 0–3 for each patient. A patient with a score of zero had no limited components, and a score of 3 indicated three limited components, similar to the lowest tertile of handgrip strength, muscle mass, as well as gait speed.

Multinomial logistic regression analyses were used to investigate the association between each of the components of sarcopenia, and the three clinical stages of cognitive impairment using SCI as the reference group. All the analyses included one unadjusted model (Model 1) and one adjusted model (Model 2) to consider potential confounding factors. Demographic features included as confounders were age, gender (female/male), education (years), and body mass index (BMI) calculated by weight (kilograms) divided by height (meters) squared. A *p*-value of <0.05 was considered significant. All statistical analyses were performed using The Jamovi Computer Software (Version 2.0.1.0) [33].

## 3 RESULTS

The final study cohort included 368 patients and consisted of 210 patients with SCI (57.1%), 95 patients with MCI (25.8%), and 63 with AD (17.1%). The distribution of the diagnoses well reflects the population referred to the memory clinic. Patient characteristics according to cognitive status are described in Table 1. The age ranged from 31 to 82 years; mean 59.0 years (SD±7.25), and 216 (58.7%) were women. The patients had, on average, 13.4 years (SD±3.1) of education and a median MMSE and MoCA score of 27 (IQR±5.0) and 24.5 (IQR±6.0), respectively.

**Table 1:** Characteristics of study participants

	ALL	SCI	MCI	AD	*p*
	(*n* = 368)	(*n* = 210)	(*n* = 95)	(*n* = 63)
Age (y)	59.0±7.25	56.9±(6.88)	60.7 (6.61)^*^^†^	63.4 (6.83)^*^^†^	<0.001
Female, *n* (%)	216 (58.7)	135 (64.3)	41 (43.2)^*^	40 (63.5)	0.002
Education (y)	13.4±3.13	13.9±2.93	12.5±3.41^*^	13.1±3.05	0.003
Education	0.002
Primary school, *n* (%)	41 (11.2)	14 (6.8)	20 (21.1)	7 (11.1)
Secondary school, *n* (%)	121 (33.2)	64 (30.9)	33 (34.7)	24 (33.2)
University, *n* (%)	203 (55.6)	129 (62.3)	42 (44.2)^*^	32 (50.8)
MMSE^‡^	27±5.00	28±3.00	27±4.00^*^^†^	22±6.75^*^^†^	<0.001
MoCa^‡^	24.5±6.0	26±4.0	23.5±5.0^*^^†^	16±7.0^*^^†^	<0.001
BMI (k*g*/*m*^2^)	26.7±4.92	26.8±4.74	28.2±5.46^†^	24.5±3.78^*^^†^	<0.001
FMI, females (k*g*/*m*^2^)	10.9±3.95	10.7±3.89	12.4±4.43^†^	9.99±3.35^†^	0.073
FMI, males (k*g*/*m*^2^)	8.13±3.28	8.05±3.31	8.86±3.39^†^	6.67±2.47^†^	0.026
Probable sarcopenia *n* (%)	33 (9.6)	13 (6.6)	14 (15.9)^*^	6 (10.3)	0.049
Sarcopenia *n* (%)	12 (3.5)	4 (2.0)	5 (5.8)	3 (5.4)	0.136
Muscle strength (*n* = 342)
HGS, females (kg)	23.9±6.35	24.8±6.39	20.8±5.95^*^	23.9±5.77	0.003^*^
HGS, males (kg)	38.2±9.64	38.51±8.83	38.12±11.19	37.05±8.42	0.791
Low HGS, *n* (%)	33 (9.6)	13 (6.6)	14 (15.9)^*^	6 (10.3)	0.049^*^
Muscle mass (*n* = 282)
FFMI, females (k*g*/*m*^2^)	15.5±1.79	15.7±1.78	15.8±1.98	14.7±1.40^*^	0.007^*^
FFMI, males (k*g*/*m*^2^)	19.0±1.99	19.29±1.82	19.02±2.29	18.13±1.58^*^	0.041^*^
Low FFMI, *n* (%)	86 (30.5)	47 (28.5)	18 (25.4)	21 (45.7)^*^	0.045^*^
Physical performance (*n* = 366)
GS (m/s)	1.28±0.24	1.33±0.23	1.21±0.25^*^	1.22±0.23^*^	<0.001^*^
Slow GS, *n* (%)	12 (3.3)	4 (1.9)	7 (7.4)^*^	1 (1.6)	0.034^*^

Values are presented as mean (SD) or median (IQR) ^‡^for continuous variables, or numbers (%) for categorical variables. SCI, subjective cognitive impairment; MCI, mild cognitive impairment; AD, Alzheimer’s disease; MMSE, Mini-Mental State Examination; MoCa, Montreal Cognitive Assessment; BMI, body mass index; FMI, fat mass index; HGS, handgrip strength; FFMI, fat free mass index; GS, gait speed. Low Handgrip strength: Female <16, Male <27 kg [6]. Low FFMI: Female <15 Male <17 kg/m^2^ [29] Slow Gait speed: ≤0.8 m/s [6]. The following data were missing: Education missing total 3 (SCI 3), MMSE missing total 103 (SCI 61, MCI 29, AD 13), MoCa missing total 10 (SCI 2, MCI 3, AD 5), BMI missing total 48 (SCI 27, MCI 14, AD 7), FMI missing total 86 (SCI 45, MCI 24, AD 17), Probable Sarcopenia missing total 26 (SCI 14, MCI 7, AD 5), Sarcopenia missing total 29 (SCI 14, MCI 8, AD 7), HGS 26 missing total (SCI 14, MCI 7, AD 5), FFMI missing total 86 (SCI 45, MCI 24, AD 17), GS missing total 2 (SCI 2). ^*^indicates these characteristics are significantly (*P* < 0.05) different between MCI and SCI groups, or between SCI and AD groups. ^†^indicates the characteristics are significantly different between MCI and AD groups.

### 3.1 Prevalence of sarcopenia, reduced handgrip strength, low fat free mass index, and low gait speed

**Fig. 2:**
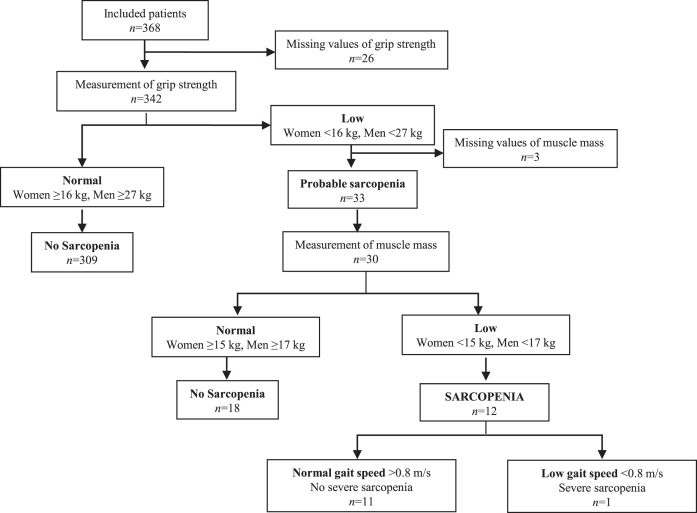
Algorithm for diagnostic assessment of sarcopenia according to EWGSOP2.

Overall, 9.6% of all patients had low handgrip strength, 30.5% had low FFMI, and 3.5% had slow gait speed, with no apparent differences between SCI, MCI, and AD (Table 1). Thus, 33 (9.6%) patients were identified with probable sarcopenia, and 12 (3.5%) patients were diagnosed with sarcopenia based on the EWGSOP2 suggested algorithm (Fig. 2). One patient was classified as severely sarcopenic.

**Fig. 3:**
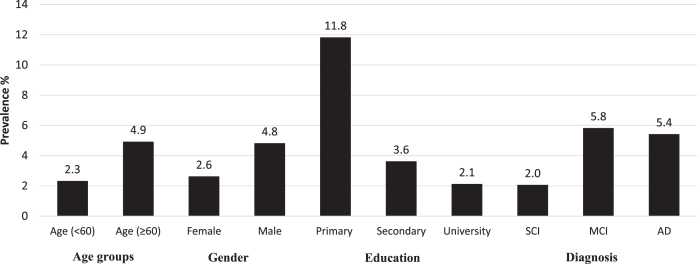
Prevalence of sarcopenia by demographic factors and cognitive status.

The prevalence of probable sarcopenia was 6.6%, 15.9% and 10.3% in SCI, MCI, and AD, respectively (Table 1). The difference between SCI and MCI was statistically significant. Further, the prevalence of sarcopenia was 2.0% in the SCI group, and increased to 5.8% in MCI and 5.4% in AD. However, these differences were not statistically significant. The sarcopenic patients had significantly lower scores of MMSE and MoCA than the non-sarcopenic patients (Supplementary Material). The prevalence of sarcopenia by demographic factors and cognitive status is described in Fig. 3. Patients with sarcopenia were characterized as expected by poorer grip strength, lower FFMI, slower gait speed, but also with fewer years of education. No significant differences in age, sex, BMI, and FMI were identified between patients with and without sarcopenia. We also did not observe any statistical difference between men and women regarding gait speed across SCI (difference = –0.0396; *p* = 0.309), MCI (difference = –0.0218; *p* = 0.680), or AD group (difference: –0.0772; *p* = 0.200).

### 3.2 The association between the components of sarcopenia and cognitive impairment

The associations between the components of sarcopenia and the various cognitive impairment diagnoses are shown in Tables 2 and 3. Table 2 displays the associations with the sarcopenia components first as continuous variables, and then categorized into normal and low based on cut-off values. The results from the multivariable analysis adjusted for cofounders showed a significant association between gait speed and cognitive impairment. Gait speed, assessed as a continuous variable, was significantly associated with both MCI and AD (using SCI as reference) after adjusting for age, education, sex, and BMI, i.e., MCI: OR 0.24 (95% CI 0.06–0.90), and AD: OR 0.12 (95% CI 0.03–0.60) (Table 2). The association between muscle strength and muscle mass and cognitive impairment disappeared in model 2 after controlling for age and education. Similar results were shown when the diagnostic component score was calculated according to the tertile model (Supplementary Material).

**Table 2:** Associations of grip strength, FFMI, and gait speed with cognitive impairment: i.e., OR for each exposure was calculated for MCI and AD separately with SCI as reference

MCI group				AD group			
Model 1		Model 2		Model 1		Model 2	
	OR (95% CI)	*p*	OR (95% CI)	*p*	OR (95% CI)	*p*	OR (95% CI)	*p*
Muscle strength
HGS, continuous (*n* = 342)	1.01 (0.99–1.04)	0.387	0.99 (0.95–1.02)^a^	0.412	0.99 (0.96–1.02)	0.454	1.01 (0.97–1.06)^a^	0.519
Female HGS (*n* = 197)	0.89 (0.83–0.96)	0.001^*^	0.95 (0.88–1.02)^b^	0.144	0.98 (0.92–1.04)	0.444	1.01 (0.94–1.09)^b^	0.734
Males HGS (*n* = 145)	0.99 (0.96–1.03)	0.821	1.00 (0.96–1.05)^b^	0.888	0.99 (0.94–1.03)	0.541	1.03 (0.96–1.09)^b^	0.406
Normal HGS (*n* = 309)	1.00 (Ref)		1.00 (Ref)		1.00 (Ref)		1.00 (Ref)
Low HGS (*n* = 33)	2.66 (1.29–5.94)	0.017^*^	1.64 (0.69–3.90)^a^	0.261	1.62 (0.59–4.48)	0.349	0.79 (0.24–2.60^a^	0.698
Muscle mass
FFMI, continuous (*n* = 282)	1.12 (1.00–1.25)	0.042^*^	1.06 (0.89–1.26)^c^	0.510	0.85 (0.73–0.98)	0.026^*^	0.80 (0.64–1.00)^c^	0.050
Female FFMI (*n* = 164)	1.03 (0.82–1.28)	0.820	1.25 (0.95–1.66)^d^	0.115	0.66 (0.49–0.89)	0.007^*^	0.78 (0.57–1.09)^d^	0.143
Male FFMI (*n* = 118)	0.93 (0.76–1.14)	0.498	0.96 (0.77–1.20)^d^	0.735	0.73 (0.54–0.98)	0.033^*^	0.79 (0.57–1.09)^d^	0.148
Normal FFMI (*n* = 196)	1.00 (Ref)		1.00 (Ref)		1.00 (Ref)		1.00 (Ref)
Low FFMI (*n* = 86)	0.85 (0.45–1.61)	0.621	0.69 (0.35–1.34)^c^	0.271	2.11 (1.08–4.13)	0.029^*^	1.53 (0.74–3.16)^c^	0.250
Physical performance
GS, continuous (*n* = 366)	0.11 (0.04–0.33)	<0.001^*^	0.21 (0.06–0.74)^a^	0.015^*^	0.13 (0.04–0.44)	0.001^*^	0.13 (0.03–0.53)^a^	0.005^*^
Normal GS (*n* = 354)	1.00 (Ref)		1.00 (Ref)		1.00 (Ref)		1.00 (Ref)
Slow GS (*n* = 12)	4.06 (1.16–14.2)	0.029^*^	2.16 (0.51–9.06)^a^	0.294	0.86 (0.09–7.50)	0.863	1.21 (0.13–12.57)^a^	0.869

SCI, subjective cognitive impairment; MCI, mild cognitive impairment; AD, Alzheimer’s disease; HGS, handgrip strength; FFMI, fat free mass index; GS, gait speed; OR, odds ratio; CI, confidence interval. Low Handgrip strength: Female <16, Male <27 kg [6]. Low FFMI: Female <15 Male <17 kg/m^2^ [29]. Slow gait speed: ≤0.8 m/s [6] ^a^Adjusted for age, education, sex, and BMI. Model 1 is crude model, and model 2 is adjusted model. ^b^Adjusted for age, education, and BMI. ^c^ Adjusted for age, education, and sex. ^d^Adjusted for age and education.

**Table 3:** Associations of the aggregated score and cognitive impairment; OR for each exposure was calculated for MCI and AD separately with SCI as reference

MCI group				AD group			
Model 1		Model 2		Model 1		Model 2	
	OR (95% CI)	*p*	OR (95% CI)	*p*	OR (95% CI)	*p*	OR (95% CI)	*p*
Aggregated (*n* = 274)
Score, continuous	0.79 (0.67–0.94)	0.008^*^	0.87 (0.73–1.05)^b^	0.139	0.68 (0.55–0.84)	<0.001^*^	0.77 (0.61–0.97)^b^	0.026^*^
Number of limited
components
0	1.0 (Ref)		1.0 (Ref)		1.0 (Ref)		1.0 (Ref)
1	2.14 (1.06–4.33)	0.034^*^	1.64 (0.78–3.44) ^b^	0.188	6.20 (2.32–16.60)	<0.001^*^	4.29 (1.45–11.92)^b^	0.005^*^
≥2	2.74 (1.36–5.51)	0.005^*^	1.99 (0.95–4.17) ^b^	0.069	6.32 (2.32–17.19)	<0.001^*^	3.89 (1.36–11.10)^b^	0.011^*^

SCI, subjective cognitive impairment; MCI, mild cognitive impairment; AD, Alzheimer’s disease; OR, odds ratio; CI, confidence interval. ^a^Adjusted for age, education, and BMI. Model 1 is crude model, and model 2 is adjusted model. ^b^Adjusted for age and education.

When aggregated, the number of limited components were significantly associated with AD (Table 3). Compared to the reference group (without limited components), the adjusted OR of AD was 4.29 (95% CI 1.45–11.92) for having one limited component, and OR 3.89 (95% 1.36–11.10) for those with two or more limited components. In addition, we observed a significant association of the number of limited components with MCI in the univariate model. However, this association became non-significant after adjustment for relevant confounding factors.

## 4 DISCUSSION

In this cross-sectional study conducted within a specialized memory clinic, focusing on outpatients, probable and confirmed sarcopenia prevalence was 9.6% and 3.5%, respectively. The prevalence increased with the degree of cognitive impairment. However, these differences were only significant between SCI and MCI for probable sarcopenia. The observed results could be explained by the variations in age, gender, and education among the different groups. It is also plausible that patients with SCI engage in more physical activity, which may increase muscle mass and enhance cognitive function through positive effects on the cardiovascular and metabolic systems. Due to the relatively low number of observations this is difficult to speculate upon. When the sarcopenia components were assessed separately, gait speed was associated with cognitive impairment. One limited component of sarcopenia, i.e., either reduced muscle mass, handgrip strength, or gait speed, was associated with an increased risk of cognitive impairment. Still, the risk did not increase further by two or more limited components. This could imply that already mild physical limitation characterize younger and mildly cognitively impaired populations.

There is a scarcity of data on sarcopenia in early cognitive decline. Most previous data are generated from older populations and patient cohorts. The reported sarcopenia prevalence in this study of 3.5% is lower than previously has been reported. However, this difference likely corresponds to the younger age of the current population [7, 21, 22], since sarcopenia is strongly associated with high age [34]. Compared to other memory clinics, we investigate considerably younger patients (mean age of 59 years) due to its specialization [21, 35]. However, different assessment protocols might also contribute to inconsistencies between studies since the cut-off values and assessment techniques chosen are crucial when identifying people with sarcopenia. Over recent years various sarcopenia diagnostic formulas have been used. A global consensus for diagnostic criteria of sarcopenia is lacking. Although cut-offs need to be adapted to ethnic groups [36], an agreement on a general definition is essential. Although the prevalence of sarcopenia was low, probable sarcopenia was registered in 9.6% of the population. Given the low mean age, this might be an important finding since probable sarcopenia, according to Cruz-Jentoft et al. [6], is enough to consider interventions to prevent further decline. Further, in real-world memory clinics, both patients and doctors might not be initially aware of the presence of sarcopenia. However, it is imperative for doctors to explore this aspect, considering its impact on not just cognitive function but also physical health both of which are essential to ensure optimum patient care [37]. Further research is needed to clarify how sarcopenia and cognitive impairment and dementia are related, by exploring the relationship between cognition and each of the specific components of sarcopenia, i.e., muscle strength, muscle mass, and physical performance.

No association between muscle mass nor handgrip strength and cognitive impairment was found in this study. Still, several previous studies have found an association between weaker grip strength and cognitive impairment [17, 19, 20]. This inconsistency might as well be due to the younger age of the current study sample [38], whereas methodological differences in assessing the handgrip strength also have to be considered. Patients with MCI and AD had a slower gait speed than those diagnosed with SCI. In addition, slow gait speed was associated with cognitive impairment. These results are consistent with previous findings [16, 17, 21].

Due to the expected low prevalence of sarcopenia in this young group of memory impaired patients, we decided to also determine the number of relatively limited sarcopenia components (the lowest tertile), and how this number related to cognitive function. Interestingly, by using tertile defined cut-offs (in contrast to diagnose-specific cut-offs) we noticed that >1 limited sarcopenia component associated with MCI (unadjusted) and AD (adjusted for age and education) with SCI as reference. This finding also supports previous reports on probable relationships [14, 15], and further indicates that the components of sarcopenia associate with cognitive impairment. Thus, measurement of gait speed might contribute to identify individuals at elevated risk for dementia in clinical settings [39]. Nevertheless, due to the cross-sectional nature of this study, we cannot definitively assert the direction of the relationship, as it could potentially operate in the opposite direction as well.

Not all patients with MCI progress to dementia, but it is essential to detect those at risk [13]. A combination of MCI and slow gait speed seems to be a strong predictor of further cognitive deterioration into AD, and is proposed as a subgroup of MCI named motoric cognitive risk syndrome [39]. Moreover, a newly derived sarcopenic index [37] showed that gait speed was the single most important indicator of cognitive decline in community-dwelling older adults [40]. We may speculate that for clinical practice, it could be important to identify motoric cognitive risk syndrome in order to possibly delay or decrease the risk of further cognitive decline with lifestyle interventions [41]. Repeatedly, studies show that cognitive and physical impairments appear combined [42, 43], i.e., sedentary behavior, low physical activity, and low muscle strength is associated with risk of dementia in observational studies [44–46]. Impaired glucose and lipid metabolism may represent a mediating mechanism. Insulin is known as an important neuromodulator and several studies have observed an association between insulin resistance and both faster rates of cognitive decline and increased risk of memory impairment in older populations [47]. Low muscle mass and inactivity are major contributors to insulin resistance.

Cognitive impairment may lead to lifestyle changes with decreased physical activity causing loss of muscle mass and muscle strength, i.e., a risk for sarcopenia [38], whereas the opposite is likely as well. On the other hand, several studies report that low physical activity and sarcopenia predict cognitive decline indicating that exercise, physical activity, and resistance training might have the potential to postpone or slow cognitive decline into MCI and dementia [48, 49], which further conveys this complex relationship. We do not know if there is an accelerated body degenerative or fragilization process in people with cognitive decline that makes them have sarcopenia earlier. Possible mechanisms include inflammation and peripheral neurodegeneration related to energy metabolism, oxidative stress, glucose metabolism, tissue repair, and growth factor response, all of which are important in protecting from cognitive and physical decline [50].

To the best of our knowledge, this is the first study in a real-world memory clinic cohort that has examined the prevalence of sarcopenia among patients with cognitive deficits. Moreover, few studies have examined the relationship between the defining components of sarcopenia and cognitive impairment among patients with clinically diagnosed SCI, MCI, and AD. The thorough phenotyping by clinicians using many tests and biological markers is a strength of the study, as well as the standardized test protocol and the blinded testing procedure. Another strength of the study is the use of the updated EWGSOP2 algorithm to diagnose sarcopenia, reflecting the latest scientific and clinical evidence in this area [6].

However, the study has its limitations. First, we reported only the actual results of our study, and the tertile may vary in different population groups. To further elaborate on this limitation, future studies should develop their own cut-off values based on the population they are studying. Second, the sample contained missing values for various variables. A large part of the missing values was due to the outbreak of the SARS Cov-2 pandemic, as some of the clinical activities temporarily had to be paused. This interfered with BIA evaluation and measurement of handgrip strength, for instance. Nevertheless, it did not affect the distribution between the different diagnoses of cognitive impairment, as the dropout rate was about the same in the three groups. Third, some critical factors such as nutrition, physical activity level, mental health, and comorbidity were not adequately evaluated in this study, and should be accounted for in future studies. Fourth, this study did not have a control group with normal cognition; instead, the SCI group was used as the reference group. Finally, the cross-sectional design of this study limits the possibility of determining any causality. Longitudinal studies are needed to explore the causal relationship between the components of sarcopenia and cognitive impairment.

### 4.1 Conclusion

In summary, this study applied the diagnostic components of sarcopenia (muscle strength, muscle mass, and physical performance) to a real-world specialized memory clinic cohort of patients. The findings revealed correlations between the components of sarcopenia and cognitive impairment across various stages within the spectrum of dementia. The study confirmed previous findings that slow gait speed is associated with cognitive impairment in populations from a clinical setting. However, more investigation is needed in this field, especially from a perspective of using longitudinal designs and including biomarkers. Such knowledge may support clinical practice for early detection of risk factors for both sarcopenia and cognitive deteriorations. It could be hypothesized, and an objective of future intervention trials, that sarcopenia treatment may offer a delay of the cognitive decline.

## Supplementary Material

Supplementary Material

## Data Availability

The GEDOC database is continuously updated. The GEDOC Steering Committee is open to requests from external researchers for data collected in this study. Requesters will be asked to submit a study protocol, including the research question, planned analysis, and data required. Committee will evaluate this plan (i.e., relevance of the research question, suitability of data, quality of proposed analyses, planned/ongoing GEDOC analyses, and other matters) on a case-by-case basis and provide the data or reject the request. Shared data will encompass the data dictionary and de-identified data only. Any analysis will be conducted in collaboration with the GEDOC Steering Committee. Access is subject to the GEDOC legal framework. An access agreement will be prepared and signed by both parties.
