# Supplementary material for: Association of Sarcopenia and Its Defining Components with the Degree of Cognitive Impairment in a Memory Clinic Population
Source: J Alzheimers Dis. 2023 Nov 7;96(2):777–88. doi: 10.3233/JAD-221186 (PMC10657697; doi:10.3233/JAD-221186)
Supplement: Supplementary Material [file jad-96-jad221186-s001.pdf]

Supplementary Material

Association of Sarcopenia and Its Defining Components with the Degree of Cognitive Impairment in a Memory Clinic Population

Supplementary Table 1. Characteristics of patients with and without sarcopenia.

All (n=339)	**No sarcopenia**	**Sarcopenia**	*p*
No. of subjects (%)	327 (96.5)	12 (3.5)	
Clinical cognitive status			0.136
SCI, n (%)	164 (98)	4 (2.0)	
MCI, n (%)	82 (94.3)	5 (5.7)	
AD, n (%)	53 (94.6)	3 (5.4)	
Age (y)	58.7 ± 7.19	62.2 ± 7.57	0.261
Female, n (%)	189 (57.8)	5 (41.7)	0.267
Education	13.6 ± 3.15	11.8 ± 2.92	0.048*
BMI (kg/m^2)	26.8 ± 4.99	25.0 ± 2.82	0.204
FMI (kg/m^2)	9.69 ± 3.92	9.73 ± 3.34	0.970
MMSE‡	27 ± 4.00	23.5 ± 4.00	0.015*
MoCa‡	25 ± 6.00	22.5 ± 6.5	0.016*
HGS (kg)	30.6 ± 10.3	15.6 ± 6.63	<0.001*
FFMI (kg/m^2)	17.1 ± 2.58	15.3 ±0.86	0.016*
GS (m/s)	1.29 ± 0.24	1.09 ± 0.21	0.006*

Valeus are presented as mean (SD) or median (IQR)‡ for continuous variables, or numbers (%) for categorical variables. BMI, body mass index; FMI, fat mass index; MMSE, Mini-Mental State Examination; MoCa, Montreal Cognitive Assessment; HGS, handgrip strength (kg); FFMI, Fat free mass index (kg/m^2); GS, gait speed (m/s)

The following data were missing: Education missing total 3 (No 3), BMI missing total 32 (No 32), FMI missing total 64 (No 64), MMSE missing total 96 (No 92, Yes 4), MoCa missing total 10 (No 10), FFMI missing total 64 (No 64), GS missing total 2 (No 2).

Supplementary Table 2. Associations of grip strength, FFMI, and gait speed, assessed as tertiles, with cognitive impairment, i.e., OR was calculated for each component separately with the lowest tertile as reference.

	MCI group				AD group			
	Model 1		Model 2		Model 1		Model 2	
	OR (95% CI)	*p*	OR (95% CI)	*p*	OR (95% CI)	*p*	OR (95% CI)	*p*
**HGS** (*n*=342)								
Lowest	1.0 (Ref)		1.0 (Ref)		1.0 (Ref)		1.0 (Ref)	
Medium	0.55 (0.30-1.01)	0.052	0.60 (0.30-1.20) [a]	0.150	0.92 (0.45-1.88)	0.824	1.07 (0.48-2.40) [a]	0.862
Highest	0.48 (0.26-0.90)	0.021*	0.65 (0.32-1.33) [a]	0.237	0.68 (0.33-1.44)	0.315	1.17 (0.50-2.75) [a]	0.714
**FFMI** (*n*=282)								
Lowest	1.0 (Ref)		1.0 (Ref)		1.0 (Ref)		1.0 (Ref)	
Medium	0.51 (0.25-1.06)	0.072	0.62 (0.29-1.33) [b]	0.221	0.26 (0.12-0.60)	0.002*	0.35 (0.15-0.83) [b]	0.018*
Highest	1.06 (0.54-2.08)	0.859	1.56 (0.75-3.25) [b]	0.232	0.36 (0.16-0.81)	0.013*	0.61 (0.25-1.47) [b]	0.270
**GS** (*n*=366)								
Lowest	1.0 (Ref)		1.0 (Ref)		1.0 (Ref)		1.0 (Ref)	
Medium	0.80 (0.45-1.42)	0.439	0.98 (0.50-1.90) [a]	0.950	0.55 (0.28-1.07)	0.076	0.57 (0.27-1.23) [a]	0.153
Highest	0.28 (0.15-0.54)	<0.001*	0.39 (0.18-0.85) [a]	0.017*	0.27 (0.13-0.55)	<0.001*	0.23 (0.09-0.54) [a]	<0.001*

SCI, subjective cognitive impairment; MCI, mild cognitive impairment; AD, Alzheimer's disease; HGS, handgrip strength; FFMI, fat free mass index; GS, gait speed; OR, odds ratio; CI, confidence interval.

Handgrip strength in each group:

Lowest tertile: Female <21, Male <35; Medium: Female 21-26.9, Male 35-41.9; Highest tertile: Female >27, Male ≥42 kg.

FFMI in each group:

Lowest tertile: Female <14.6, Male <18.3; Medium: Female 14.6-15.9, Male 18.3-19.89; Highest tertile: Female ≥16, Male ≥19.9 kg/m^2.

Gait speed in each group:

Lowest tertile <1.2; Medium tertile 1.2-1.39; Highest tertile >1.4 m/s.

[a] Adjusted for age, education, and BMI

[b] Adjusted for age and education

Supplementary Table 3. The prevalence of sarcopenia by age of patients

	Young patients (<=70 years)			Older patients		
	SCI	MCI	AD	SCI	MCI	AD
N	4	4	2	0	1	1
Percentage	2.1	4.8	4.1	0	25	14.3
